# A world unglued: simultanagnosia as a spatial restriction of attention

**DOI:** 10.3389/fnhum.2013.00145

**Published:** 2013-04-17

**Authors:** Kirsten A. Dalrymple, Jason J. S. Barton, Alan Kingstone

**Affiliations:** ^1^Department of Psychological and Brain Sciences, Dartmouth CollegeHanover, NH, USA; ^2^Institute of Cognitive Neuroscience, University College LondonLondon, UK; ^3^Department of Medicine (Neurology), Ophthalmology and Visual Sciences, University of British ColumbiaVancouver, BC, Canada; ^4^Department of Psychology, University of British ColumbiaVancouver, BC, Canada

**Keywords:** Bálint syndrome, simultanagnosia, object-based attention, space-based attention, vision

## Abstract

Simultanagnosia is a disorder of visual attention that leaves a patient's world unglued: scenes and objects are perceived in a piecemeal manner. It is generally agreed that simultanagnosia is related to an impairment of attention, but it is unclear whether this impairment is object- or space-based in nature. We first consider the findings that support a concept of simultanagnosia as deficit of object-based attention. We then examine the evidence suggesting that simultanagnosia results from damage to a space-based attentional system, and in particular a model of simultanagnosia as a narrowed spatial window of attention. We ask whether seemingly object-based deficits can be explained by space-based mechanisms, and consider the evidence that object processing influences spatial deficits in this condition. Finally, we discuss limitations of a space-based attentional explanation.

Look around at your surroundings: the room you are sitting in, the pictures on the walls, the desk in front of you, and the words on this page. Your visual system is processing a wealth of information about those objects, such as their size, shape, color, and location. Although your visual system fits all these elements together to create a coherent picture of your world, patients with simultanagnosia view the world in a chaotic manner (Rafal, 2001). These patients can see only one object at a time and sometimes only pieces of objects, unaware that they are locked on just one component of a larger form. What they *can* see cannot be located in space, likely because they see nothing else that can provide a reference point to situate objects in the world (Rafal, 2003).

The term simultanagnosia was coined by Wolpert (1924), who described it as an ability to perceive the individual elements of a scene, without the ability to synthesize the overall meaning of the scene. Farah (1990) later specified the existence of dorsal (as opposed to ventral^1^) simultanagnosia, which results from bilateral parieto-occipital damage, including the intraparietal sulcus and bilateral visuospatial white matter pathways (Chechlacz et al., 2012a), and is best described as a restriction of visual attention such that the patient is only aware of one object at a time (Rizzo and Vecera, 2002; Moreaud, 2003; Rafal, 2003). Despite intact visual acuity, the attentional deficit of simultanagnosia can be so debilitating that patients are often described as being functionally blind (Holmes and Horrax, 1919; Rafal, 1997; Kim and Robertson, 2001). Patients may see “an ant or an elephant, but only one object at a time” (Rafal, 2001, p. 122), and cannot localize these items nor use visual information to interact with them.

In an early study of a patient with Bálint syndrome, of which simultanagnosia forms an important part, Holmes and Horrax (1919) identified the simultanagnosic deficit as “visual inattention.” The patient's inability to see more than one object at a time was not due to blindness, they explained, because he had fully functioning retinas^2^. “The essential feature was his inability to direct his attention to, and take cognizance of, two or more objects that threw their images on the seeing portion of his retinae. As this occurred no matter on what parts of his retinae the images fell, it must be attributed to a special disturbance or limitation of attention … ” (Holmes and Horrax, 1919, p. 390).

There are several ways to conceptualize what attention is and how the visual system uses attention to create a coherent visual scene. The visual system is constantly flooded with information about different objects and stimuli and one must select a subset of this information for further processing. Attention is the mechanism that allows this selection to occur, but there are competing theories about what guides that selection. One type of attentional selection is object-based: attention is directed to objects that are defined based on pre-attentive segmentation in accordance with basic grouping principles (e.g., Duncan, 1984; Driver and Baylis, 1989). A second type of attentional selection is space-based: attention is directed to locations in space irrespective of objects (Posner et al., 1980). Given that visual attention is involved in creating a coherent visual representation of our world, attribution of the piecemeal perception of simultanagnosia to a faulty attentional system is not surprising, but is the attentional deficit primarily object-based, or space-based in nature? Simultanagnosia is often conceptualized as an *object-based* disorder, in that the attention involved in selecting objects or their features is compromised. An alternate account is that simultanagnosia is a *space-based* disorder of attention: if attention can only process information within one restricted location, it may be difficult to select more than one object at a time. While there is no reason why object- and space-based attentional deficits cannot both be present in this syndrome, it is worth considering whether one deficit can also account for at least a fair share of the findings usually considered as evidence for the other. For example, while the limitation of recognition to single objects in simultanagnosia could suggest reduced capacity of object-based selection, it could alternatively be conceptualized as *preserved* object-based selection, in that patients can recognize single objects, coupled with impaired space-based attention, which restricts the number of objects that can be perceived.

In this review we first summarize what is known about the neural substrates of attention, particularly with regards to simultanagnosia and a related disorder of attention, visual neglect. We then consider the case for simultanagnosia as a result of damage to an object-based attentional system, examining the evidence that simultanagnosic patients neglect whole objects and also tend to perceive objects in parts. We then review the evidence suggesting that simultanagnosic patients suffer from damage to a space-based attentional system, in particular whether it can be understood as a restricted spatial window of attention. We next ask whether apparent object-based deficits could be explained more parsimoniously by impaired space-based mechanisms. Finally, we discuss limitations to the space-based attentional framework, alternate views of simultanagnosia, and considerations for future research.

## Neural substrates of attention

Focused attention, on objects or spatial locations, is necessary for conscious perception of visual information (Neisser, 1967). In healthy individuals, “change blindness” (i.e., the inability to detect even large changes in an object or scene, Rensink et al., 1997) and the “attentional blink” (i.e., the failure to consciously perceive a stimulus that rapidly follows another stimulus, Raymond et al., 1992) are examples of how the absence of focused attention can result in a lack of awareness of features of the visual world that are in plain sight, i.e., “looking” but not “seeing” (Rizzo and Hurtig, 1987). In clinical cases of disordered attention, such as simultanagnosia and unilateral visual neglect, the failure to consciously perceive visual stimuli is pathological and provides a wealth of information about the nature of object- and space-based attention and their neuroanatomical substrates.

The relatively high prevalence of unilateral visual neglect (Stone et al., 1993; Bowen et al., 1999), which is typically considered to be a disorder of spatial attention, has allowed for a large number of investigations into the anatomy of space-based, but also object-based attention. Parallels between neglect and simultanagnosia are striking, leading some to describe simultanagnosia as “bilateral neglect” (Michel and Henaff, 2004, p. 10) though distinctions in lesion locations (Rafal, 1997) and behavioral dissimilarities suggest that this is an over simplification^3^. Neglect results from unilateral temporoparietal lesions and is defined by a failure to attend to information on the contralesional (usually left) side of space (Heilman and Valenstein, 1979). Of particular relevance to the present discussion, neglect has been conceptualized both in terms of spatial and object-specific deficits (e.g., Driver and Halligan, 1991; Behrmann and Moscovitch, 1994; Medina et al., 2008; Corbetta and Shulman, 2011; Chechlacz et al., 2012b). In the context of neglect, spatial deficits refer to inattention to viewer-centered (egocentric) spatial locations, while object-specific deficits refer to a failure to attend to object-centered (allocentric) locations (e.g., the left side of the object, irrespective of where the object is located relative to the perceiver). Spatial attention deficits in neglect have been linked to the dorsal stream network, particularly the right supramarginal gyrus, while object-centered deficits have been linked to more ventral regions including posterior inferior temporal, lateral occipital, and posterior middle/inferior temporal regions (Medina et al., 2008; Chechlacz et al., 2012b), suggesting a link between these types of attention and those neuroanatomical regions.

In contrast to neglect, simultanagnosia is reported in a much smaller proportion of neurological cases. The scarcity of patients and the variability in lesion locations between patients makes it difficult to determine the precise neuroanatomical regions that are affected in the disorder, or their relationship to object- versus space-based deficits. Despite these challenges, studies using techniques such as lesion overlap, voxel-based morphometry (VBM), and diffusion tensor imaging (DTI), have been used to address this issue. A recent study with a group of simultanagnosics combined these techniques to determine that bilateral damage to the medial occipito-parietal junction, the cuneus, and the inferior intra-parietal sulcus, in addition to the underlying white matter tracts, is critical for producing simultanagnosia (Chechlacz et al., 2012a). The authors attributed the white matter damage to deficits in processing speed, while damage to the occipito-parietal junction was attributed to the impaired ability to represent multiple items together and attend to global aspects of compound forms. Consistent with these interpretations, a study of a patient with transient simultanagnosia due to posterior cortical atrophy linked the medial occipito-parietal regions and the cuneus to the integration of multiple elements into a global Gestalt (Himmelbach et al., 2009), an ability that is notably impaired in simultanagnosia (Karnath et al., 2000; Shalev et al., 2004, 2007; Huberle and Karnath, 2006; Dalrymple et al., 2007). Himmelbach and colleagues used functional magnetic resonance imaging (fMRI) to determine that these areas were active when the patient successfully identified the global level of hierarchical stimuli. Riddoch et al. (2010) interpreted this finding as evidence that these areas are involved in controlling the spatial window or scale of visual attention. Along similar lines, Michel and Henaff (2004) attributed their simultanagnosic patient's deficits, which resulted from bilateral and relatively symmetrical lesions between the upper calcarine fissure and the occipito-parietal sulcus, extending to white matter in the fronto-parietal areas, to a weakening and shrinkage of the attentional visual field. Thus, different behavioral trademarks in simultanagnosia have been linked to damage to different parts of the visual attentional networks, speaking to the contributions of those areas to visual attention. We now consider how certain behaviors in simultanagnosia speak to the nature of the attentional impairments that define the disorder.

## Evidence for object-based deficits in simultanagnosia

Object-based theories suggest that attention is directed to candidate objects that are then selected for further processing. Although objects are segmented pre-attentively based on basic properties like spatial proximity, contour, or color, focal attention, which has limited capacity, selects target objects for more detailed processing (Duncan, 1984). In these theories, the limitation in attentional capacity is viewed in terms of the number of separate objects that can be perceived at once (Duncan, 1984). Simultanagnosia, the inability to see more than one object at one time, is therefore understood as an abnormal limitation of this attentional capacity: “It is whole objects that are neglected, not spatially determined parts of objects; and the objects that are neglected may occupy the same spatial coordinates as an object that is seen” (Rafal, 2001, p. 128). Patients with simultanagnosia have often been studied with the aim of determining what constitutes an object because these patients are “aware of only one entity, and typically that entity is an object rather than a constituent part” (Rafal, 2003, p. 352). However, patients have been reported to both ignore some objects in their entirety (e.g., Rizzo and Hurtig, 1987; Coslett and Saffran, 1991; Humphreys and Price, 1994; Rafal, 2001) and to see other objects in parts (e.g., Luria, 1959; Karnath et al., 2000; Riddoch and Humphreys, 2004; Huberle and Karnath, 2006; Dalrymple et al., 2007).

### Unawareness of whole objects

Much anecdotal evidence from bedside testing suggests that simultanagnosia is related to inattention to objects. Patients report that it is difficult to watch television because they cannot see more than one person or object at a time. Coslett and Saffran (1991) said of their patient, “she reported watching a movie in which, after a heated argument, she noted to her surprise and consternation that the character she had been watching was suddenly sent reeling across the room, apparently as a consequence of a punch thrown by a character she had never seen.” (p. 1525). Rafal (2001) reports that when a comb and a ruler were presented to patient RX, either the comb or the ruler were perceived, but not both at the same time. This can occur even if parts of both objects occupy the same location. For example, Holmes and Horrax (1919) report that a patient was able to identify the shape of a square on a page, but when a fixation cross was added, he saw only the cross, despite the fact that the square and the cross shared the same location. Humphreys and Price (1994) reported that when a word and a picture were presented simultaneously at the same location, their two patients usually reported seeing only the picture. Because they share the same location, unawareness of the second object would not appear to reflect a spatial variability of attention, such as is present in hemineglect. Even more dramatically, patients can be unaware of objects that they are fixating. Rizzo and Hurtig (1987) describe three patients with simultanagnosia who reported the spontaneous disappearance of objects from awareness, despite the fact that eye movement recordings showed that they were still fixating the objects. They attributed this “looking but not seeing” to a failure of sustained attention to objects.

Object meaning can influence inattention to objects in simultanagnosia. Coslett and Saffran (1991) found that their patient was better at identifying two simultaneously presented words when these were components of compound words (e.g., BASE and BALL) or semantically related (e.g., HOT and COLD), than when they were unrelated. Better recognition was also found for pairs of line drawings that were semantically related (e.g., both animals) than those that were unrelated (e.g., animal and tool). In both cases the spatial locations of the stimuli remain constant, therefore pointing to object-based effects that can modulate the attentional deficit of simultanagnosia (although of course this does not demand the conclusion that the deficit itself is object-based).

### Part-based perception of objects

**Figure 1:**
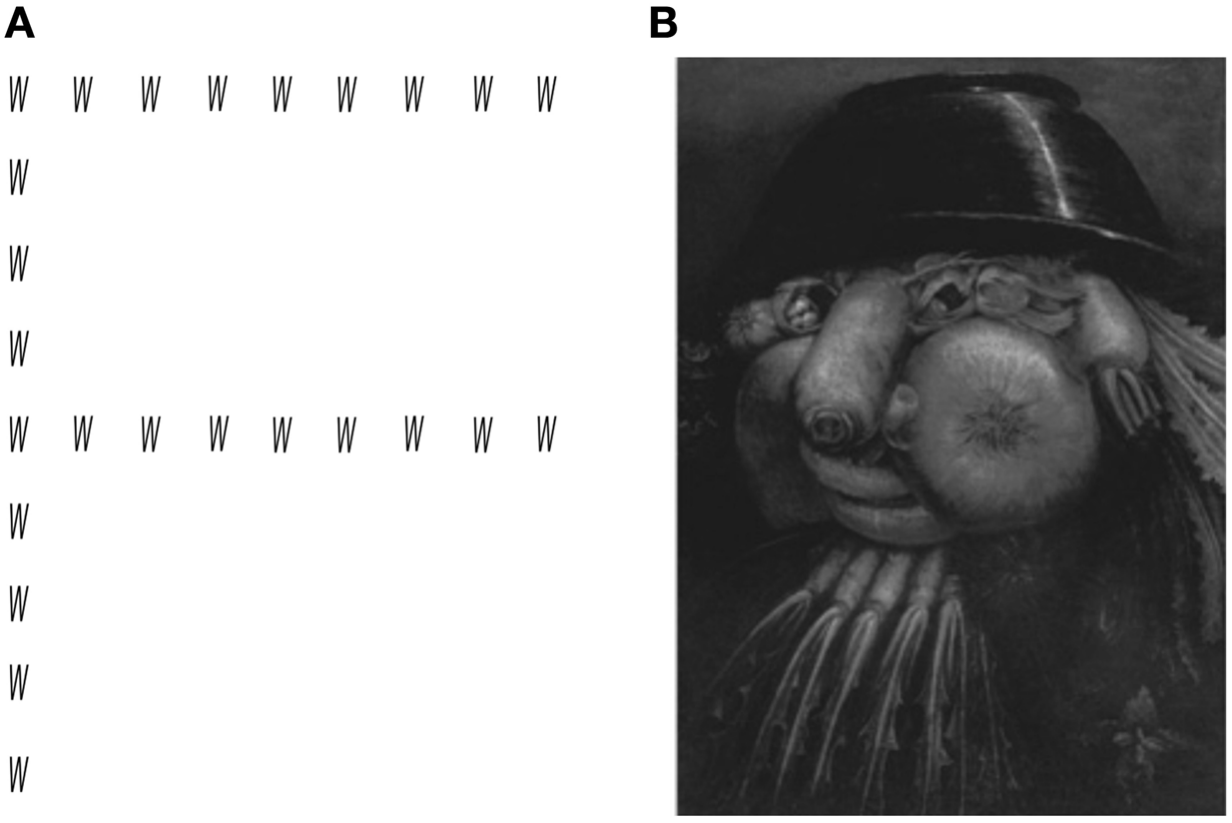
**Examples of hierarchical stimuli frequently used as stimuli in experiments with patients with Bálint syndrome, (A) global letter made up of local letters; (B) global face made up of a theme of local elements, in this case fruit.** Painting by Giuseppe Arcimboldo.

In addition to inattention to whole objects, there is evidence that patients with simultanagnosia perceive single objects in a piecemeal manner, indicating another manifestation of damage to an object-based attentional system. “Local capture” (Karnath et al., 2000) is the best demonstration of this point: patients identify the local components of an object but fail to see the global aspect of the object, even with unlimited presentation (e.g., Figure 1A; Huberle and Karnath, 2006; Dalrymple et al., 2007).

**Figure 2:**
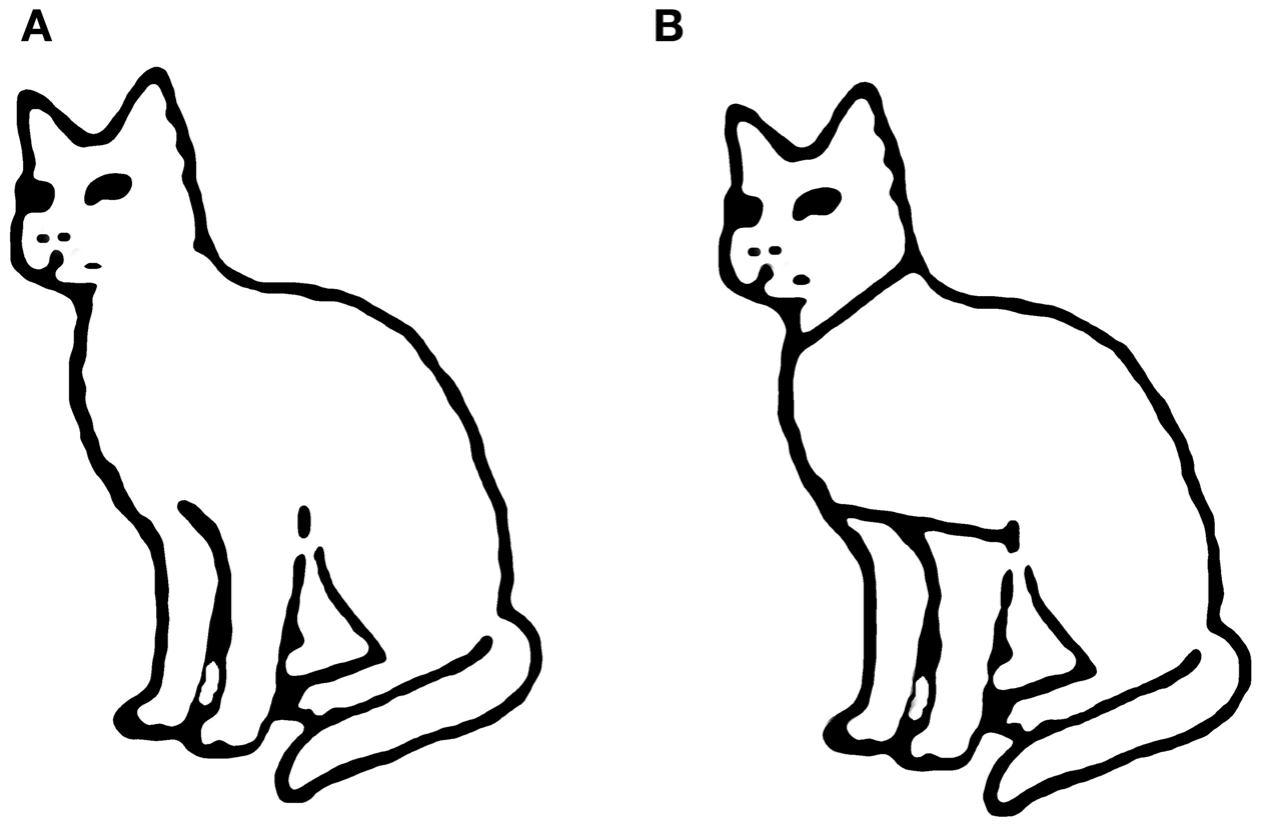
**Example of stimuli from Riddoch and Humphreys (2004) showing (A) line drawing of animate object and (B) artificial segmentation of that line drawing**.

**Figure 3:**
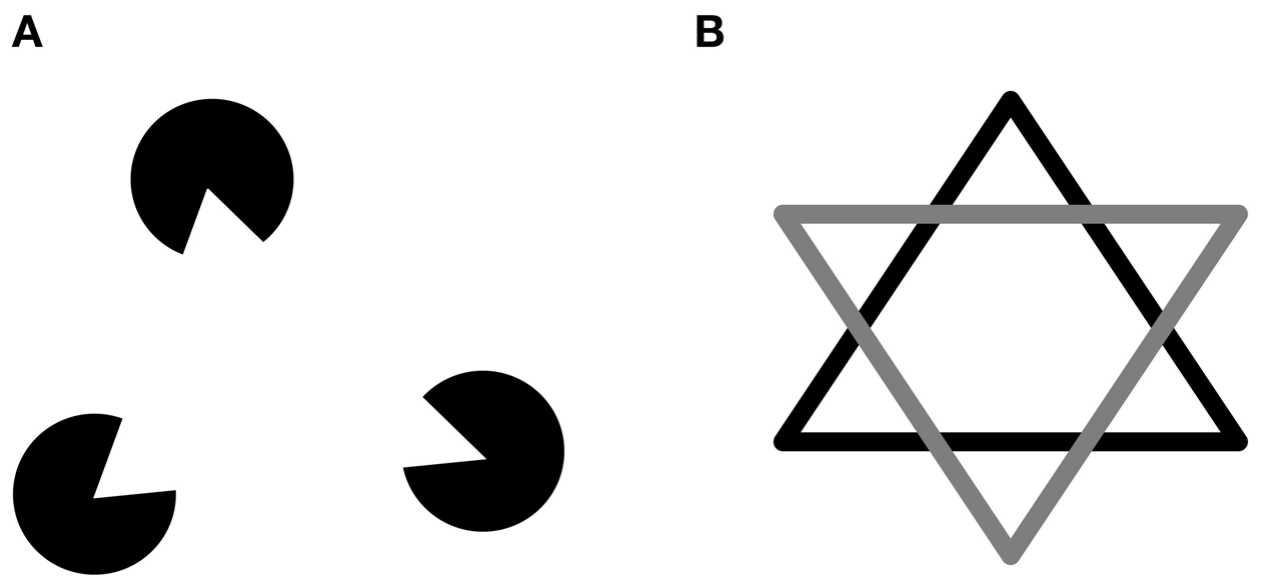
**(A)** Kanizsa (illusory) triangle; **(B)** Star of David.

The component properties of objects can influence how they are perceived. Coslett and Saffran (1991) found that their simultanagnosic patients could name words but not non-words, despite the fact that both had a similar spatial span. They suggested that words were processed as single objects, while non-word letter strings were processed as multiple objects (i.e., distinct letters). Riddoch and Humphreys (2004) reported that simultanagnosic patients were accurate at identifying objects in line drawings but had difficulty when these drawings were artificially segmented (Figure 2), despite the fact that the spatial coordinates of both types of drawings were identical. Similarly, the simultanagnosic patient SL could not make judgments about the triangular spatial relationship between three separate discs, but could do so if the solitary triangle was made explicit by adding lines connecting the discs, or surface texture to the triangle (Figure 3A; Barton et al., 2007). It has been reported that another patient did not see multiple circles until they were joined by a line, at which point he reported seeing, “two circles, resembling spectacles.” (p. 442) (Luria, 1959). When this patient saw a Star of David (Figure 3B) colored to give the impression of two overlapping triangles, he only reported one triangle, whereas when it had a single color, he saw the complete star. These types of findings are often taken as evidence regarding the properties required to strongly link disparate features into a single object. In this view, reduced object-based attention results in failure to perceive such objects when the linkage between features is weakened.

## Evidence for space-based deficits in simultanagnosia

Space-based attention is the direction of attention to particular locations (Posner et al., 1980). The role of the parietal lobes in visual attention (Lynch et al., 1977; Wurtz et al., 1982; Corbetta and Shulman, 2002; Corbetta et al., 2008) and spatial processing is clear (Mishkin, 1972; Ungerleider and Mishkin, 1982). Ungerleider and Mishkin's influential 1982 review “Two cortical visual systems” cites behavioral, electrophysiological, and anatomical evidence, including a systematic ablation study with monkeys, that segregate the processing of objects (the “what” system) and the processing of space (the “where” system) into two separate neural pathways. According to this review, the ventral stream, which extends from early visual areas through the inferotemporal cortex, is responsible processing objects, while the dorsal stream, which extends from early visual areas through the parietal cortex, is responsible for processing information about space. Goodale and Miller (1992) amended this theory to suggest that the dorsal stream is responsible for computing action (the “how” stream). More recently, these authors proposed an integrated account of object and space processing, suggesting that both visual streams have been implicated in the processing of object and space information and that both streams are influenced by attention (Milner and Goodale, 2008). Chechlacz et al. (2012a) concluded that simultanagnosia results from bilateral parieto-occipital white matter disconnections within the visuospatial attention network, thus explicitly linking simultanagnosia with current understandings of the anatomical locus of space-based attention. As discussed earlier, others have similarly linked the neural substrates of simultanagnosia to space-based behavioral deficits (Michel and Henaff, 2004; Himmelbach et al., 2009; Riddoch et al., 2010). Here we consider other behavioral evidence that links simultanagnosia to impaired processing of space.

### Space-based behavioral deficits in simultanagnosia

Feature Integration Theory (FIT) proposes that objects are created through the binding of features that occupy the same location in space (Treisman and Gelade, 1980). While features may be processed pre-attentively, focused attention is necessary to combine features into perception of an object. Patients with simultanagnosia experience a large number of illusory conjunctions, incorrectly binding features from different objects (Friedman-Hill et al., 1995; Robertson et al., 1997; McCrea et al., 2006; Coslett and Lie, 2008). According to FIT, the unusually large number of illusory conjunctions in simultanagnosia is evidence of impaired spatial processing or limited spatial attention, along with mislocalization of object features. A series of experiments that demonstrates this spatial deficit in simultanagnosia was conducted with patient RM (Wojciulik and Kanwisher, 1998). RM experienced frequent illusory conjunctions with an explicit binding task, in which he was asked to report the identity of a word written in colored text while ignoring a distractor word written in white. To perform the task correctly one must bind the color at a certain location with the word at that location. RM performed poorly on this task.

More direct evidence of poor spatial processing is evident from other tasks. RM was also greatly impaired at reporting the spatial location of items on a screen in either absolute terms or relative to other objects, regardless of whether items were presented sequentially or simultaneously (Friedman-Hill et al., 1995). In another example, he could not report whether a word was located at the top, middle, or bottom of a rectangle (Robertson et al., 1997).

Despite these explicit spatial deficits, simultanagnosic patients may still correctly process spatial information implicitly. Though RM could not report the location of a word in a rectangle, he showed a spatial Stroop effect when reading the word, being slower to read the word “UP” when it appeared at the bottom rather than the top of the rectangle (Robertson et al., 1997). Hence spatial information was being processed to create congruency effects between the verbal and spatial properties of the stimulus. RM also showed normal spatial orienting in a Posner cueing paradigm (Egly et al., 1995), and demonstrated implicit feature binding on a classic Stroop test, being faster to name the color of text if it spelled the name of that color than if it spelled the name of a different color (Wojciulik and Kanwisher, 1998). This occurred even though he could not explicitly report which text color was associated with which word. The basis of such implicit spatial processing remains unclear, but Cinel and Humphreys (2006) suggest that it reflects initial binding of color and shape based on co-location and grouping, with poor spatial attention leading to decay of bound features prior to explicit report. Alternatively, Robertson et al. (1997) suggest that implicit processing of space in simultanagnosia may reflect preserved information from retinotopic maps in striate cortex. Finally, Rizzo and Hurtig (1987) propose the existence of an attentional bottleneck in simultanagnosia that allows spatial information to be processed partially, sufficient to guide eye movements but not enough for conscious awareness.

### Simultanagnosia as a reduced “spatial window of attention”

Feature integration theory is one way of explaining the relationship between spatial attention and object perception, but there are many ways of conceptualizing spatial attention itself (Cheal et al., 1994). One way that is particularly relevant to discussions of simultanagnosia is the idea of attention as a “spotlight” (Shulman et al., 1979; Posner et al., 1980; Tsal, 1983). This proposes that, like a spotlight, attention can be moved and directed to various locations in space. The spotlight is often described as flexible: it can zoom out to cover a large spatial area, or zoom in to cover a smaller spatial area (Humphreys, 1981). Furthermore, just as a spotlight becomes brighter when the beam is narrowed, attentional acuity may increase as the spatial extent of an attended area is reduced in size (Humphreys, 1981).

The concept of simultanagnosia as reflecting a restricted window (or “spotlight”) of attention is apparent from descriptions used in the literature. Michel and Henaff (2004) discuss the simultanagnosic deficit in terms of a shrinkage of the attentional visual field. Bay (1953) suggested that simultanagnosia could be accounted for by “shaft vision” that prevented the patient from seeing the whole picture, or similarly as a “peripheral constriction,” not unlike “viewing [a] picture through a diaphragm” (p. 545, 546). Thaiss and de Bleser (1992) suggested that their patient TK suffered from a rigidly reduced visual window, as TK perceived whole objects, parts of objects, and even multiple small objects if they appeared to fit within a narrow visual window. Tyler (1968) also referred to the visual deficit in his patient as “shaft vision” (p. 166), but suggested that there was some dynamic flexibility to this constriction. The “effective” field of his patient always included the central 2°, but could expand up to 20° in some circumstances, though it tended to fatigue within 10–30 s. Shalev et al. (2004) have also reported priming results consistent with flexible reduction of a spatial attentional window in simultanagnosia. Seeing a large solid letter improved the ability to recognize the global aspect of a subsequent hierarchical letter of the same size and location. Shalev et al. suggested that the restricted window of attention was temporarily widened by the prime, allowing explicit processing of the global hierarchical letter.

The suggestion that the anomalously constricted window of attention maintains some flexibility in simultanagnosia is also highlighted by an experiment contrasting hierarchical letter and face stimuli (Figure 1). Like other patients with simultanagnosia, SL showed local capture with hierarchical Navon letter stimuli, naming the small local elements but not the global aspect (Dalrymple et al., 2007). However, SL demonstrated “global capture” with hierarchical Arcimboldo faces (e.g., a face made up of fruits and vegetables), reporting the global percept of a face more than its local components. SL did not report seeing the local elements unless they were interpreted in terms of the global face. For example, when viewing a face made up of vegetables, she reported seeing vegetables “on the head” and the face of “a goddess” (Dalrymple et al., 2007). Similar examples of global capture can occur in more natural encounters. Rafal (2001) described a woman who could see his face but could not discern whether he was wearing glasses. The ability of simultanagnosic patients to see the global levels of facial stimuli may require an expanded window of attention in the presence of stimuli with highly salient global properties, in which there is strong linkage between component features (Moscovitch et al., 1997; Farah et al., 1998). Furthermore, one interpretation of the consequent failure to see the local elements when the global form is perceived is that expansion of the window occurs at the expense of attentional acuity: they can see a face, but cannot resolve its details^4^. This interpretation is consistent with some introspective statements by patients: For example, GB said, “… my visual field is like a cone that I can extend or shorten. I spend most of my time with a very short visual field concentrating on only one or two things at a time … At times, I have to extend my visual field … This is difficult, because … detail is lost with the extended field, and sometimes everything blends into one” (pers. communication, May 2010).

There are data showing that information can still be processed to a degree outside of the restricted window of attention (that supports conscious awareness of stimuli). This can account for some types of implicit processing. Even when patients cannot name the global level of hierarchical letters, they can still show congruency effects, being faster to name the local letter if it is the same as the global letter (Karnath et al., 2000; Shalev et al., 2007). Similarly, patients can show normal cueing by peripheral stimuli in a Posner cueing paradigm (Egly et al., 1995) and normal performance on pop-out search tasks (Karnath et al., 2000).

A restricted window of attention may also explain why single objects are sometimes perceived in parts. Thaiss and de Bleser (1992), like others (e.g., Bay, 1953; Tyler, 1968; Michel and Henaff, 2004), suggested that their patient TK may suffer from reduced spatial extent of an attentional window. When shown hierarchical stimuli, TK reported only the local elements and not the larger global picture. When asked to copy a simple line drawing, TK reproduced only the individual lines and did not show evidence of seeing the global whole. However, with line drawings of different sizes, TK showed superior performance for naming the objects in smaller drawings. The simple interpretation was that small objects fit within TK's narrowed attentional window, while the larger objects did not, so that for the latter only fragments were processed, consistent with Riddoch and Humphreys (2004) “partonomic error.” A parallel observation is that the ability to see global aspects of hierarchical letters is better when the global size is smaller (Shalev et al., 2004; Huberle and Karnath, 2006; Dalrymple et al., 2007).

Can a restricted window of attention also explain other phenomena in Bálint syndrome? These patients often have difficulty localizing objects, for example. Relevant to a narrow processing window, Tyler (1968) asked whether a patient with constricted visual fields and no other brain damage would show such spatial disorientation. Similarly, Farah (1990) pondered whether a subject seated in a dark room could determine the relative location of sequential flashes of light on a wall. In both scenarios, the issue is whether narrowing perception to one isolated object at a time would impair object localization. Indeed, Rafal (2003) proposed that this would be the case, particularly for the localization of one object relative to another (it is challenging to tell where one object is located relative to another if you can only see one of those objects at a one time). This issue was recently tested (Dalrymple et al., 2010). When viewing hierarchical letters, it should be theoretically possible to deduce the global form even with a narrow window of attention by simply moving this window over many local elements and then linking their positional information together (i.e., mentally “connecting the dots”). However, healthy subjects viewing displays with an artificially restricted visual window showed impairments in reporting the global aspect of hierarchical letters similar to those seen a simultanagnosic patient. Hence a small window of processing, restricted in either visual or attentional capacity, can impair the relative localization and spatial integration of local elements needed to infer global form.

A restricted window of attention can also account for more complex behavioral changes. We examined how the simultanagnosic patient SL scanned social scenes, and then tested whether healthy subjects viewing the scenes through a restricted visual window would show similar scanning patterns (Dalrymple et al., 2011a). While healthy subjects under normal viewing conditions consistently allocate a large number of fixations to the eyes of people in scenes (Smilek et al., 2006; Birmingham et al., 2007, 2008a,b), both SL and healthy subjects with a narrow viewing window showed an abnormally low proportion of fixations on eyes. When SL was tested again 3 years later, the proportion of fixations on eyes had increased, though it was still abnormal (Dalrymple et al., 2011b). Interestingly, a similar increase in fixations on eyes could be produced in healthy subjects by increasing the size of the viewing visual window, suggesting that one possible mechanism of spontaneous recovery is expansion of a restricted window of attention.

## Can space-based mechanisms explain object-based effects?

If a space-based restriction of attention can account for some phenomena often seen with simultanagnosia, it is worthwhile to consider if it can also explain any of the findings that have previously been interpreted as evidence of object-based attentional deficits. After all, objects themselves have spatial dimensions: at a basic level, the spatial arrangement of object components can define the object (Biederman, 1987). For example, a curved tube attached to the side of a cylinder indicates a cup, while the same curved tube attached to the top of a cylinder creates a bucket (Biederman, 1987; Shalev and Humphreys, 2002); the spatial relation between these two components determines the perceived object. This illustrates a very simple scenario where object perception requires preserved representation of spatial relationships of parts, but there are more complex ways in which the processing of space is important for object perception.

With this relationship between space and object perception in mind, space-based theories can explain how whole objects are neglected in simultanagnosia. Robertson and colleagues (1997) argue that damage to a space-based system can account for findings that seem to indicate object-based effects, such as those demonstrating that patients with simultanagnosia extinguish objects based on object properties, independent of space (e.g., Humphreys et al., 1994). They argue that in the case of damaged spatial input, objects can be selected based on object properties and that this explains why some objects are extinguished at the expense of others in simultanagnosia.

The basic simultanagnosic phenomenon of seeing only one object at a time can be readily explained by impaired spatial selection, with preserved object processing allowing the remaining object to be identified. Riddoch and Humphreys (2004) argued that the fact that basic Gestalt principles like connectivity, axis alignment, colinearity, and closure, still affect patient performance indicates that, “the fundamental aspects of object coding continue to operate in simultanagnosia.” (p. 424). It has been argued that simultanagnosia is a misnomer, because “agnosia” implies a failure of recognition whereas patients with simultanagnosia can typically recognize the objects that they see (Rizzo and Robin, 1990). This is consistent with the conceptualization of simultanagnosic patients as “object-detectors” (Holmes and Horrax, 1919; Baylis et al., 1994). Indeed, there is some evidence of priority for object selection over spatial selection in simultanagnosia, implying preservation of the former and deficits in the latter. For example, GK was asked to read words presented at fixation, while a distractor object was presented either above or below the words. Despite the fact that the words were presented in a spatial position that favored their selection (e.g., central fixation), when he only reported one item, it was more often the picture than the word (Humphreys et al., 1994), indicating a hierarchy that shows preferred selection of salient objects over selection of spatial location.

There are theoretical accounts that explain how damage to spatial processing can disturb object perception. According to one such explanation, there are two types of object recognition systems: a spatial one that defines the object in terms of shape and location, and the other, a retinotopic representation of the object, which is activated by top-down information from the first system. Object shape and size are defined in spatial terms, being created through the combination of various locations in a spatial array. Disruption of this spatial system leaves an object's shape and size undefined (Farah, 2004), a conclusion supported by computational simulations (Mozer, 2002) and consistent with the fact that simultanagnosic patients sometimes have inaccurate perception of single objects, as when they make “partonomic” errors, mistakenly seeing parts of objects as whole objects (Riddoch and Humphreys, 2004).

## Object-based modulation of space-based deficits

Object properties may influence the competition between local and global perception in a situation where spatial attentional capacity is restricted. One such factor is familiarity with the elements. Shalev et al. (2007) showed patient GK hierarchical letters that were global English letters (familiar to GK) made up of local Hebrew letters with which he was initially unfamiliar, and then trained to recognize. At the beginning GK was quite accurate at seeing the global English letter, but with training on Hebrew letters, his performance for the global English letters declined. This finding helps explain how preserved object processing can influence allocation of spatial attention. Shalev et al. describe attention as a “horse race” between object components: whichever component attracts attention first will pull attention to that spatial area or scale. This suggests that the allocation of attention to space critically depends on the identity of the object itself and its components. Although healthy subjects would still be able to report both local and global components, with a latency advantage to the winner of this race, the limited attentional capacity of simultanagnosic patients instead means that only one component emerges from this competition. In the example above, the increased saliency conferred by familiarity enhances the processing at the relevant spatial scale and modulates the likelihood of GK reporting the global letter.

Another example of such modulation is seen in studies of the “two object cost” (Duncan, 1984). Healthy subjects are better at making two judgments about a single object compared to a single judgment about two objects. One might predict that this effect would be heightened in simultanagnosic patients, because they see only one object at a time and would be impaired at making judgments about multiple objects. In support of this prediction, Cooper and Humphreys (2000) showed that GK was better at making within-object spatial judgments relative to between-object spatial judgments, an observation also made later in SL (Barton et al., 2007). GK had to judge the relative height of two vertical bars, which he did better when the two bars were connected at their base to make a “U”-like single object. However, this effect was in turn modulated by the familiarity of the resulting single object, as it was not found with less letter-like configurations, and could even be influenced by instructions that suggested the presence of single or multiple objects in the display (Shalev and Humphreys, 2002). Configurations of lines and dots that corresponded to a strong known stimulus category (e.g., “face”) tended to show better spatial processing than those that did not (e.g. “oval with two circles”), suggesting an important contribution of stored object representations to the effect. Shalev and Humphreys (2002) explained these findings in terms of a restricted spatial zoom lens of attention. They suggested that patients can move the narrowed lens from object to object to make the two-object judgments, yet struggle to expand that zoom lens to go from object parts to whole objects in order to make within-object judgments. Stored representations may facilitate the expansion of the zoom lens, explaining why patients show an advantage for within-object judgments with familiar objects and between-object judgments with unfamiliar objects.

Preserved object-based attention can also *direct* space-based attention in patients with simultanagnosia (Humphreys and Riddoch, 2003). Simultanagnosic patient GK was asked to report the presence of one or two shapes placed on either side of fixation. The objects varied in the strength of their perceptual grouping, such that one shape was “closed” (i.e., a square made up of lines with cornered edges) and the other was “open” (i.e., a square made up of lines without corned edges). As in an earlier study (Humphreys et al., 1994), closed shapes were reported more often than the open shapes regardless of spatial position. To determine whether this object bias could direct spatial attention, Humphreys and Riddoch (2003) used the closed and open shape stimuli as cues for a letter identification task. They briefly presented GK with the closed and open shapes on either side of fixation, followed by letters at the same spatial locations. GK reported the letter at the location of the non-extinguished closed shape more frequently than the letter at the location of the extinguished open shape. However, GK could not report where the letter was located relative to either fixation or the preceding cuing shape, suggesting that the preserved object processing could direct spatial attention but could not promote conscious spatial processing. Humphreys and Riddoch suggested that, with damage to the spatial selection system as in simultanagnosia, preserved object processing exerts more influence on selection, controlling the weaker, spatial attention system.

In another example, Kim and Cave (2001) performed a study demonstrating what they call “object-based location selection” (p. 620), the influence of object features on the spatial selection of stimuli. They presented three letters: a target letter at fixation, flanked by two distractor letters. One of the distractor letters matched the target letter on a feature (color) while the other did not. Subjects were asked to identify the target letter, and then respond to a probe that appeared immediately following the letter display. Simultanagnosic subjects were faster to respond to the probe when it appeared at the location of the distractor that had the same color as the target, demonstrating spatial selection acting on the location of stimuli grouped on the basis of a shared object feature.

Finally, in the reverse direction, impaired spatial attention can *disrupt* the perception of objects. Smooth pursuit is the tracking of objects with the eyes. With smooth pursuit, the object remains constant, but its spatial coordinates constantly change. A reduced capacity for the processing of space can therefore interfere with the ability to perceive the object as it moves through space. Rafal (1997) reports this phenomenon in simultanagnosic patient MB as she attempted to follow a pen with her eyes: “she lost the pen after pursuing it only a few degrees, and even though it was still only a few degrees from fixation, it vanished. She stopped pursuing it with her eyes because she could no longer see it.” (p. 344). A simple explanation of this event is that the pen had moved outside the spatial confines of a narrow window of attention.

## Limitations of the restricted window of attention model

Some simultanagnosic effects cannot be easily explained by a reduced spatial window of attention, or at least suggest further modifications of the latter. The illusory conjunctions mentioned before are one example (Robertson et al., 1997). One might expect that a reduced window would allow processing of a single object falling within it and promote the correct binding of the object's features, yet patients frequently show binding errors. These illusory conjunctions can also occur in healthy subjects with brief viewing under conditions of divided attention. Patient RM showed fewer illusory conjunctions when stimuli were presented sequentially in the same location, than when stimuli were presented simultaneously in different locations, suggesting more problems with binding in space than across time. However, it may also be that the processing of stimuli at two locations requires some expansion of the narrowed attentional window, which has a consequent reduction in spatial resolution of attention in simultanagnosic patients, allowing binding errors to occur. This argument is similar to that used above to explain why local elements can fail to be perceived in “global capture” (Dalrymple et al., 2007) and it is consistent with the introspective statements by patients like GB (see section Simultanagnosia as a reduced “spatial window of attention,” above).

Another effect that may be difficult to account for under a spatial window theory is the spontaneous disappearance of objects that are being fixated (Rizzo and Hurtig, 1987). Rizzo and Hurtig speculate that this reflects cortical fatigue, which prevents sustained processing of stimuli to a level of conscious awareness, consistent with Pavlov's hypothesis that the visual deficits in simultanagnosia were related to “low tonus of excitation” in visual cortex (Pavlov, 1955, p. 609). This hypothesis is supported by Luria's (1959) finding that the administration of the stimulant caffeine improved simultanagnosic symptoms. For example, with tachistoscopic presentation of two simultaneous figures, performance improved from seeing both figures on 0 of 30 trials to seeing both on 12 of 30 trials. While Luria's results could be explained by an expansion of the window of attention with caffeine administration, it is more difficult to explain how single objects that are fixated may disappear. One possibility is that with cortical fatigue, the window of attention closes entirely. Another explanation is that information processing *within* the window of attention can be fatigued.

A restricted window of attention may have difficulty explaining the finding that simultanagnosic patients show normal cuing effects during a Posner cuing paradigm (Egly et al., 1995). However, studies of normal attention show that stimuli outside the central focus of attention can attract attention (Posner et al., 1980), and there is evidence that this still occurs in simultanagnosia. For example, Robertson et al.'s (1997) spatial Stroop experiment demonstrated implicit processing of location information in simultanagnosia, despite an absence of explicit location knowledge. These findings would suggest that visual stimuli outside of the confines of the narrowed spatial window of attention are not subject to complete failure of representation, but rather a weaker degree of representation than normal.

Finally, can a restricted window of attention explain why patients with simultanagnosia cannot see more than one object at time even when those objects appear to occupy the same spatial location, as with the overlapping figures test (e.g., Poppelreuter tests, Luria, 1978; Rafal, 2001, 2003)? In considering this question it is important to note that the spatial overlap of items is never complete, and it is apparent that subjects are often aware of the presence of other items besides the one they report. For example, with overlapping line drawings, GB described not only seeing a single object but also other lines that did not make sense to him. With great effort he eventually named all but one object of the overlapping figures (Dalrymple et al., 2013).

## Summary

Although our review has focused on contrasts and interactions between object- and space-based mechanisms, there are other hypotheses regarding potential processing deficits in simultanagnosia. The framework of the Integrated Competition Hypothesis (Duncan et al., 1997) has generated suggestions that processing resources are depleted in simultanagnosia, resulting in all or nothing competition between objects (Jackson et al., 2009). Others contend that an inability to disengage from attended stimuli prevents the perception of new stimuli (Farah, 1990), though recent tests of this idea have failed to support it (Clavagnier et al., 2006; Dalrymple et al., 2009). Yet others have suggested that impaired object perception in simultanagnosia results from an inability to combine preserved space and object information (Coslett and Lie, 2008). Coslett and Lie also raised the possibility that discordant findings and models of simultanagnosia may reflect the existence of distinct subtypes. They propose that one subtype is due to an impairment of early visual attention, while a second is related to a later impairment of binding between object and space information (e.g., Coslett and Chatterjee, 2003). In this classification, a restricted visual window in simultanagnosia would qualify as an early stage impairment of visual attention.

Naturally, it is not necessarily the case that these different proposals are mutually exclusive: attention is complex and multi-faceted, and the attentional defect in simultanagnosia may mirror this complexity. Indeed, it seems likely that both object- and space-based deficits will be found to co-exist in most patients, just as patients with hemineglect from unilateral lesions can show both object- and space-based deficits (Driver and Halligan, 1991; Behrmann and Moscovitch, 1994). However, in the interests of parsimony it is important to consider how many observations and experimental findings can be accommodated by a single conceptual framework. The proposal of a limited spatial window of attentional processing has a long history in our concepts of simultanagnosia, (e.g., Bay, 1953) and recent modeling work with restricted windows of visual processing in healthy subjects shows that it has considerable explanatory power for many behavioral effects in simultanagnosia (e.g., Dalrymple et al., 2009, 2010, 2011a,b, 2013). In future, understanding the interactions between preserved object perception, impaired object attention, and disrupted spatial attention will likely provide even more sophisticated insights into the behavioral puzzle that is simultanagnosia.

### Conflict of interest statement

The authors declare that the research was conducted in the absence of any commercial or financial relationships that could be construed as a potential conflict of interest.

## Footnotes

^1^Ventral simultanagnosia results from left inferior occipito-temporal damage (Farah, 1990) and is less frequently reported in the literature. Although we make this distinction here, for simplicity we will use the general term of “simultanagnosia” interchangeably with “dorsal simultanagnosia” throughout this review.

^2^Retinal function in simultanagnosia can be assessed by perimetry in the absence of distractors and with highly salient (i.e., bright) stimuli. Simultanagnosia is distinguished from neglect by the fact that it lacks the spatial bias typical of the latter.

^3^See Rizzo and Vecera, 2002, for a more complete review of the relationship between simultanagnosia and visual neglect.

^4^An alternate explanation may be the effects of reduced attentional capacity on global/local competition, so that only the stronger of the local or global percept is seen, and the weaker one not.
